# COVID-19 Lethality in Sub-Saharan Africa and Helminth Immune Modulation

**DOI:** 10.3389/fimmu.2020.574910

**Published:** 2020-10-08

**Authors:** Luis Fonte, Armando Acosta, Maria E. Sarmiento, María Ginori, Gissel García, Mohd Nor Norazmi

**Affiliations:** ^1^Department of Parasitology, Institute of Tropical Medicine “Pedro Kourí”, Havana, Cuba; ^2^School of Health Sciences, Universiti Sains Malaysia, Kelantan, Malaysia; ^3^Department of Teaching, Polyclinic “Plaza de la Revolución”, Havana, Cuba; ^4^Department of Medical Genetic, Hospital “Hermanos Ameijeiras”, Havana, Cuba

**Keywords:** Sub-Saharan Africa, helminth immune modulation, SARS CoV-2, COVID-19, lethality

The acronym COVID-19 (Coronavirus Disease 2019) identifies the human disease caused by the Severe Acute Respiratory Syndrome Coronavirus-2 (SARS-CoV-2) (1). Since the outbreak of COVID-19 in Wuhan city, China, in December 2019, it has rapidly spread through 185 countries in all continents (2). SARS CoV-2 infection, and its related disease, is now a major health problem, with 23,057,288 infected individuals and 800,906 deaths confirmed worldwide as of August 24, 2020 (3).

The natural progression of SARS CoV-2 infection is extremely variable. It ranges between an asymptomatic course or mild clinical expression, which generally occurs in children and healthy adults, and the development of pneumonia and severe multi-organ failure, more frequent in the elderly and in patients of chronic diseases. This broad spectrum of clinical expression is the consequence of another one at immunological level: SARS CoV-2 infection activates innate and adaptive immune responses that, in the most frequent and benign of evolutions, lead to the containment of viral replication and recovery and, in the most unfavorable of sequences, can stimulate an intense pulmonary inflammatory reaction that, leading to more severe complications, can end in death (4).

SARS-CoV-2, unlike its close genetic relative SARS CoV, has the ability to infect and reproduce in the upper respiratory tract (5). There, type I/III interferons, tumor necrosis factor alpha (TNF-α-) interleukin-1 (IL-1), IL-6, and IL-18, among other components of the innate immunity, control the infection in the majority of the individuals (6). However, if SARS-CoV-2 passes through that first control and spreads, along the conducting airways, to the alveoli, it can replicate there more rapidly, causing pneumonia and other severe clinical complications (7).

Severe COVID-19 evolution is associated with an increase in the proportion of Th1 and Th17 cells, with their corresponding cytokines IFN-γ, IL17, IL-23, and TNF-α (8). At the same time, there is an activation of the inflammatory CD14+CD16+ monocytes, with an amplified production of cytokines, such as IL-6, and chemokines, such as CC- chemokine ligand 2 (CCL2), CCL3 and CXC- chemokine ligand 10 (CXCL10) (8, 9). The triggering of these cellular types, and the release of their mediators, leads to an increase of inflammation, vascular permeability and leakage with severe lung damage (8).

COVID-19 has shown significant differences in its lethality rate between continents, regions and countries (3). Of them, the more notable is the higher rates registered in economically developed regions with robust health systems, such as Europe and the United States, compared to countries having poor economies and insufficient health services, in particular, almost all the nations that constitute the Sub-Saharan Africa (SSA) (Table 1) (3). Some factors, or combinations of them, have been mentioned to explain the unexpected evolution: diagnostic test unavailability, age and genetic background of the population, mutational variations of SARS-CoV-2 in relation with geographic settings, environmental temperature and humidity non-favorable for viral replication, BCG vaccination policies and endemicity of other infections (10–12). Here, we hypothesize the possible role of helminth immune modulation in the low COVID-19 lethality in SSA.

**Table 1:** COVID-19 lethality rates in Europe, United States and Sub-Saharan Africa as of August 23, 2020.

**Region**	**Confirmed cases**	**Deaths**	**Lethality (%)**
Europe	3,970,890	216,478	5.45
United States	5,567,217	174,246	3.12
Sub-Saharan Africa	959,311	18,897	1.96

***Source**: (3)*.

***Confirmed Case**: person positive by Polymerase Chain Reaction (PCR) test for SARS CoV-2*.

***COVID-19 death**: a COVID-19 death is defined as a death resulting from a clinically compatible illness in a probable or confirmed COVID-19 case, unless there is a clear alternative cause of death that cannot be related to COVID-19 disease (e.g., trauma). There should be no period of complete recovery between the illness and death*.

In 2009, Hotez and Kamath, in a landmark paper analyzed the striking connection between living conditions and prevalence of Neglected Tropical Diseases (NTDs), linking the world's greatest concentration of poverty with helminth infection prevalence in SSA region (13). In this region, “73% of the population lives on <US$2 per day, the most common NTDs, such as the soil-transmitted helminth infections, schistosomiasis, lymphatic filariasis and onchocerciasis, affected more than 500 million people” (13). For example, “of the world's 207 million estimated cases of schistosomiasis, 93% occur in SSA (192 million)” (13). Since then, little has changed in that part of the planet.

The “equilibrium” occurring in individuals chronically infected with helminths is the result of hundreds of millions of years of host-parasite coevolution. That prolonged interaction has led to the development of defensive responses by the human hosts and to the achievement of complex immune modulatory means by the helminths. The host protective responses against helminths, which are multicellular and large organisms, include wound repair mechanisms, which reduce the tissue damage that these parasites may cause as they move through body organs.

The cellular damage resulting from helminths migration through tissues is the major stimulus of the innate immunity against those parasites, as danger associated molecular patterns (DAMPs) are released and induce the production of cytokine alarmins (IL-25, IL-33, and thymic stromal lymphopoietin -TSLP-) by epithelial cells (14). IL-25 and IL-33 trigger the production of IL-4, IL-5 and IL-13, the principal mediators of type 2 responses, by type 2 innate auxiliary cells (14). On the other hand, TSLP limits IL-12 production by dendritic cells, the main promoter of type 1 responses (15).

For controlling the helminth infections, the adaptive immunity of the host usually develops type 2 immune responses, including the development of Th2 cells and the release of cytokines such as IL-4, IL-5, and IL-13 (16). This host-helminth interaction has, at least, two additional outcomes: (i) the classical and best-known down-regulation of type Th1 and type Th17 responses (and its related cytokines IL-12, IFN-γ, IL17, IL-23, TNF-α) by the Th2 cytokines (16, 17) and (ii) the helminths limitation of both host type1 and type 2 responses by enhancing FOXP3+ T regulatory cells, B regulatory cells and alternatively activated macrophages (AAMs) activities, which together cause the release of regulatory cytokines such as IL-10 and transforming growth factor (TGF-β) (18).

The modulation by helminths of the immune responses of their hosts has relevant clinical and epidemiological consequences: increased susceptibility to some infections, decreased frequency and intensity of allergic, autoimmune and inflammatory diseases, inadequate responses to vaccines and, as is possible in the case of SARS-CoV-2 infection, may inhibit the inflammatory processes that characterize infection by other microorganisms (17).

Helminths modulation has the ability to suppress inflammatory responses present during infection by protozoon, bacteria and virus: (i) when *Plasmodium falciparum* infection occurs in an individual infected with helminths, the effects of pro-inflammatory cytokines (IFN-γ and TNF-α) that characterize severe forms of malaria are attenuated by the action of anti-inflammatory mediators (IL-10 and TGF-β) and, consequently, decrease the chances of developing severe inflammatory conditions, including cerebral malaria (19); (ii) mice infected by *Nippostrongylus brasiliensis* showed increased susceptibility to *Mycobacterium tuberculosis*. Apparently, AAMs with impaired killing capacity in a less inflammatory Type 2 pulmonary milieu function as a mycobacteria reservoir (20); (iii) *Trichinella spiralis* infection limits inflammatory pulmonary damage induced by influenza virus in mice (21).

Nevertheless, and analyzing the helminth-virus relationship from a more holistic perspective, it is necessary to mention that helminths can enhance anti-viral mechanisms leading to a better control of viral load. Two examples: (i) during helminth infection IL-4 can expand and condition virtual memory CD8+ T cells (T_VM_ cells) for more rapid CD8 responses against subsequent cognate antigen encounter. Apparently, immunity against helminths has evolved a safety mechanism through induction of highly responding T_VM_ cells to counterbalance anti-inflammatory effects related to type 2 immunity on the development of effective antiviral responses (22); (ii) mice infection by the rodent roundworm, *Heligmosomoides polygyrus*, significantly reduce pulmonary lung damage and viral load following intranasal infection with respiratory syncytial virus. Interestingly, those effects were independent of adaptive immune responses because protection was lost in germ free mice, denoting a possible role of intestinal microbiota (23).

Taking into account the arguments described above, it is plausible to consider other factors, such as the inhibition of inflammatory processes by regulatory mechanisms induced by helminths, to provide an explanation to the low lethality of COVID-19 in SSA. Interestingly, and probably in connection with it, the historical data relating to SARS-CoV and Middle East respiratory syndrome-CoV epidemics reveal that these viruses caused very limited health problems, if any, in Sub-Saharan countries (24).

In a very recent paper, Bradbury et al., suggested that immune modulation by helminths could reduce the human resistance to SARS CoV-2 infection. Nevertheless, they called upon the research community to investigate whether helminth co-infection with COVID-19 could influence the pandemic spread through the helminth endemic regions of the world (25). Here, contrary to the opinion by Bradbury et al., we argue that helminth coinfection, in conjunction with at least part of the factors mentioned above, may be related to the low lethality of COVID-19 in SSA.

Furthermore, and looking ahead, we believe that helminth modulation on both type 1 and 2 immunity should be an important factor to consider during the design and evaluation of vaccines against SARS CoV-2 in those countries. The requirements of triggering type 1 responses for controlling viral replication and the development of type 2 immunopathology events observed during challenge experiments in animal models immunized with some coronavirus vaccine candidates support that reflection (26).

## Author Contributions

All authors listed have made a substantial, direct and intellectual contribution to the work, and approved it for publication.

## Conflict of Interest

The authors declare that the research was conducted in the absence of any commercial or financial relationships that could be construed as a potential conflict of interest.
